# Eyes on the Pupil Size: Pupillary Response During Sentence Processing in Aphasia

**DOI:** 10.3390/brainsci15020107

**Published:** 2025-01-23

**Authors:** Christina Sen, Noelle Abbott, Niloofar Akhavan, Carolyn Baker, Tracy Love

**Affiliations:** 1 Joint Doctoral Program in Language and Communicative Disorders, San Diego State University/University of California San Diego, San Diego, CA 92182, USA; csen7960@sdsu.edu (C.S.); nabbott2@uw.edu (N.A.); nakhavan77@gmail.com (N.A.); careybkr@gmail.com (C.B.); 2 Department of Speech and Hearing Sciences, University of Washington, Seattle, WA 98195, USA

**Keywords:** aphasia, semantic cue, sentence processing, pupillometry

## Abstract

Background/Objectives: Individuals with chronic agrammatic aphasia demonstrate real-time sentence processing difficulties at the lexical and structural levels. Research using time-sensitive measures, such as priming and eye-tracking, have associated these difficulties with temporal delays in accessing semantic representations that are needed in real time during sentence structure building. In this study, we examined the real-time processing effort linked to sentence processing in individuals with aphasia and neurotypical, age-matched control participants as measured through pupil reactivity (i.e., pupillometry). Specifically, we investigated whether a semantically biased lexical cue (i.e., adjective) influences the processing effort while listening to complex noncanonical sentences. Methods: In this eye-tracking while listening study (within-subjects design), participants listened to sentences that either contained biased or unbiased adjectives (e.g., venomous snake vs. voracious snake) while viewing four images, three related to nouns in the sentence and one unrelated, but a plausible match for the unbiased adjective. Pupillary responses were collected every 17 ms throughout the entire sentence. Results: While age-matched controls demonstrated increased pupil response throughout the course of the sentence, individuals with aphasia showed a plateau in pupil response early on in the sentence. Nevertheless, both controls and individuals with aphasia demonstrated reduced processing effort in the biased adjective condition. Conclusions: Individuals with aphasia are sensitive to lexical–semantic cues despite impairments in real-time lexical activation during sentence processing.

## 1. Introduction

Understanding spoken sentences as they unfold in real time is an intricate process, given that the listener must be able to access multiple levels of language (i.e., phonetics, semantics, syntax, etc.) and coordinate information across the different levels to achieve comprehension. Despite these complex processes, language comprehension appears seamless and is rapidly performed by neurotypical adults. However, individuals who have an acquired language impairment due to neural damage within language-related regions of the brain (those diagnosed with aphasia) often experience disruptions in the processes that support language comprehension.

Within the field of language processing, research has demonstrated that some individuals with aphasia (IWA) show deficits in lexical-level processing [1]. It has been argued that lexical-level processing impairments can have downstream effects on sentence comprehension abilities, especially for those sentences that do not follow the language’s typical (canonical) word order [2,3]. A large body of research has investigated the underlying source of real-time sentence processing deficits in IWA, suggesting that the slowed processing of lexical and/or syntactic components leads to breakdowns in the time course of sentence processing and, ultimately, sentence comprehension [4,5,6,7,8]. Processing models that focus on lexical-level deficits propose that delays in the activation or integration of lexical–semantic information lead to disruptions in the formation of syntactic structure building and, ultimately, sentence comprehension in IWA.

Lexical processing models have explored how lexical deficits affect sentence processing using methods that capture different aspects of real-time sentence processing (e.g., event-related potentials, eye-tracking, and priming). Some models argue that IWA have a delay in integrating semantic information (see [9,10,11,12]), while others argue that the delay is in accessing the semantic network [1,2,3,4,6]). In one study exploring the latter category (e.g., lexical access delays), Love et al. [4] proposed the Delayed Lexical Activation (DLA) hypothesis, which posits that, when lexical activation is slowed, it prevents the timely formation of syntactic structures. Without the necessary lexical–semantic information available in real time, fast processes like lexical access are disrupted, leading to comprehension breakdowns. Debates in the literature are still on-going as to how to differentiate the integration and access effects using methods that reflect the different stages and/or components of lexical processing (e.g., time course). The findings across a number of methods point to lexical-level delays underlying sentence-level processing impairments.

In the seminal study investigating the DLA, Love et al. [4] measured real-time, moment-by-moment lexical activation in IWA using a cross-modal lexical priming paradigm CMLP; see [8] (though we note here that this delay in lexical access has also been shown with other methods, such as eye-tracking while listening [13,14]). The Love et al. [4] study revealed that, unlike the age-matched controls who showed the immediate activation of the noun (‘wrestler’) upon hearing it, IWA had a 400 ms delay in activation (see (1) below).
objectsubjectverb(1)The audience liked [the wrestler_i_] [that the parish priest condemned_i_] for foul language.

While not the focus of this study, another interesting finding is related to the fact that the sentences tested were object-relative constructions. To understand these sentences, the listener must link the fronted direct object (‘wrestler’) to its syntactically licensed position (verb offset, ‘condemned’; noted with the subscript ‘i’) in order to understand who is doing what to whom (‘priest condemned wrestler’). The study also revealed that, at the offset of the verb, IWA showed a delay in activation, unlike the neurotypical control participants who showed on-time activation. The authors argued that the data supported the DLA hypothesis as IWA initially displayed the delayed lexical activation of the direct object and later in the sentence when the syntactically driven reactivation of the direct object (verb offset) is required.

Despite these lexical delays, studies have shown that impairments in lexical activation during sentence processing in aphasia can be mitigated. In the same study by Love and colleagues [4], the authors found that slowing the rate of speech input resulted in on-time initial lexical activation and re-activation at the post-verb gap site, with an improvement in final sentence comprehension. Subsequent studies by Love and colleagues explored other ways besides slowing the speech rate to mitigate delayed lexical access [13,14,15]. In a series of eye-tracking while listening (ETL) studies with a visual world paradigm, Baker and Love [14] found that adding a short silent pause after a target noun (the object of a sentence) provided the necessary time for IWA to demonstrate activation, while Baker and Love [15] showed lexical facilitation through attention and semantic priming tasks targeting the noun of interest prior to the onset of the sentence [14,15].

Of interest to the current study, research has also shown that lexical activation may be modulated by the lexical–semantic relationships between sentence constituents. Akhavan et al. [13] used semantic cues to explore whether lexical activation can be facilitated in IWA and age-matched controls. In this ETL study, lexical activation was measured while participants listened to sentences and viewed images on a screen that corresponded to lexical items in the sentence. Eye-tracking was used to track the location of gazes over time as a proxy measure for lexical activation during sentence processing. Akhavan et al. [13] presented participants with sentences that either contained an adjective that was semantically biased (i.e., venomous in sentence (2a)) or unbiased (i.e., voracious in sentence (2b)) toward the upcoming noun (i.e., snake). While listening to sentences, participants would see a visual display with four images. Three of the images were of the nouns in the sentence (i.e., eagle, snake, and bear) and the other image was an unrelated noun (i.e., cat).

(2a) The eagle saw the **venomous** snake_i_ that the bear cautiously encountered_i_ underneath the narrow bridge.
(2b) The eagle saw the **voracious** snake_i_ that the bear cautiously encountered_i_ underneath the narrow bridge.

The results from Akhavan et al. [13] demonstrated that, for age-matched controls, the semantically biased adjective (seen in (2a)) resulted in the faster lexical activation of the target noun (‘snake’). The researchers suggested that the biasing adjective (‘venomous’) reduced the degree of semantic interference from the competing images (i.e., ‘eagle’ and ‘bear’), allowing ‘snake’ to be more readily selected from the visual competitors. In contrast to the results for age-matched controls, IWA did not show faster lexical activation in the semantically biased adjective condition. Instead, IWA showed a previously established pattern of delayed lexical access. Thus, the gaze data suggest that IWA were unable to use semantic cues in a timely fashion to boost the lexical activation of the subsequent noun. Yet, as previously discussed, the time course of lexical activation is only one component of lexical processing, and the gaze data are limited in measuring overt looking patterns.

The current study seeks to use a related measure, pupillometry, that is more sensitive to subtle processing demands which may reflect the initial engagement of the semantic network. While pupillometry has been successfully used to capture the lexical–semantic processing at the single word level. Here, we use pupillometry to gauge IWA’s moment-by-moment sensitivity to semantic information during real-time sentence-level processing.

### 1.1. Pupillary Measures of Cognitive Load During Sentence Processing

In the present study, we explore whether semantic cues (such as those used in Akhavan et al., [13]) give rise to richer lexical representations and reduce the processing demands that are required for lexical activation. An eye-tracking method known as pupillometry was used to measure processing (cognitive) demands, or the amount of cognitive effort required for processing.

Pupillometry is defined as the measurement of the change in pupil diameter in millimeters. Researchers have found that the task-evoked responses of the pupil (TERPs) are closely tied to cognitive processes initiated in the locus coeruleus, which modulates systems such as memory, attention, and arousal [16,17]. Therefore, the pupil dilation response is considered an index of cognitive effort and can be used to continuously measure changes in cognitive effort during sentence processing as semantic and syntactic information is accessed and integrated [18,19,20]. These pupillary response effects have been shown to be sensitive to sentence complexity during auditory sentence processing in neurotypical populations and IWA [18,21,22]. In addition to syntactic complexity, researchers have also shown that the pupillary response is a reliable index of lexical and syntactic ambiguity [23], case marking [24], prosodic incongruency [19], and grammatical gender [25], to name a few.

Early pupillometry research analyzed pupillary responses in pre-defined stimulus windows; snapshots within a sentence, e.g., [23]. More recent work, however, has sought to capitalize on the continuous nature of pupillometry to better capture the changes in response over time; see, for example, [26,27]. This approach also allows for the collection of pupillary data simultaneously with gaze data, e.g., [21]. Thus, this paper capitalizes on the fine-grained and dynamic aspects of pupillometry to examine how the pupillary response changes as a sentence unfolds over time, which provides insights into the effort and timing of accessing the lexical–semantic network during sentence processing. Using this approach, we explore whether neurotypical control participants and IWA can use semantic information to reduce the processing effort at the lexical and sentence levels.

### 1.2. Pupillometry in Aphasia

Although the usage of pupillometry in aphasia research is limited, some studies have examined pupil dilations in IWA in response to word-level semantic tasks [28], syntactically complex sentences [18], and other cognitive tasks such as short-term memory tasks [29,30]. For instance, Chapman and Hallowell [28] explored how the lexical–semantic difficulty (i.e., based on factors such as word frequency and word familiarity) of auditorily presented single words influenced pupillary responses in IWA. The IWA demonstrated larger pupil responses (i.e., greater processing effort) for difficult words (lower frequency—*snail*) versus easier words (higher frequency—*sheep*). The authors argued that IWA are, in fact, sensitive to lexical–semantic information during single-word processing, as evinced by their pupillary response. In a later study, Chapman and Hallowell [18] also demonstrated that pupillary reactivity to sentence complexity was shown for neurotypical control participants, but not for IWA. The authors argued that IWA are unable to adequately expend the processing effort for syntactic dependencies due to deficits in the allocation and/or capacity of cognitive resources during sentence processing.

Thus, given that IWA are sensitive to lexical–semantic information as shown in Chapman and Hallowell [28], this study explores if IWA demonstrate a reduction in processing effort by presenting semantically biased adjectives prior to a noun of interest during real-time auditory sentence processing.

### 1.3. Present Study

In the present study, pupillary responses during auditory sentence processing were measured to assess the processing effort in object-relative constructions. The focus of this paper is on the initial processing of the direct object noun phrase when it follows a semantically biased or neutral adjective.

Below, we present sentence-level data from both age-matched neurotypical control participants (AMC) and IWA, as research has shown that the processing effort increases throughout the sentence as syntactic structures are built in real time [18]. It was anticipated that, since IWA often show auditory comprehension deficits and delayed lexical level activation, they, unlike the AMC, would show no change in pupil dilation response (i.e., processing effort) throughout the course of the sentence, reflecting a failure to build the complex syntactic structure.

As stated above, we focus here on how semantic cueing affects the time course of the processing effort during initial lexical activation. Semantic cues were presented through semantically biased and unbiased adjectives preceding a target noun. The goal was to determine whether a semantically biased adjective (e.g., in (2a) ‘venomous snake’ compared to (2b) ‘voracious snake’) helps to boost the lexical–semantic access of the upcoming noun by reducing the processing effort. Based on the prior semantic biasing research, we hypothesized that the sentences with semantically biased adjectives would result in smaller pupillary responses (i.e., less processing effort) than sentences with unbiased adjectives i.e., [31] for the AMC group (see for example, [31]). In contrast, for IWA, there are three possible outcomes in response to the semantic biasing. The first outcome is no change in pupillary response. This would reflect the impaired access to the semantic information provided by the biased adjectives, resulting in an overall failure to integrate as suggested by lexical integration deficit accounts [9,10,11,12]. The second possible outcome is an immediate decrease in pupillary response upon hearing the biased adjective, similar to the AMC group. This would reflect the immediate access to the semantic representation and spared lexical–semantic processing, as supported by Chapman and Hallowell [18]. The third outcome is a delayed decrease in pupillary response in the biased adjective condition. We argue that such a delay would disrupt timely integration, thus supporting the delayed activation hypothesis [3,4,6].

## 2. Methods

### 2.1. Participants

For this within-subjects study, 11 individuals with aphasia (IWA; female = 5, M_age_ = 61.64, SD_age_ = 2.31) and 11 age-matched controls (AMC; M_age_ = 61.9, SD_age_ = 8.18) participated. For both the AMC and IWA, participants were recruited based on the following criteria: monolingual native English speaker, right-handed (pre-morbidly for IWA), no self-reported history of emotional or learning disorders and drug abuse, and normal or corrected-to-normal vision and hearing (see below) for their age range. The AMC had no history of neurological disorders or neural trauma. IWA also had no prior history of neurological disorders, with all IWA experiencing a single, left-hemisphere stroke, and were recruited at least 6 months post-onset of stroke (Table 1). The diagnosis and severity of aphasia were confirmed using standardized aphasia examinations, including the Boston Diagnostic Aphasia Examination BDAE-version 3 [32] and the Western Aphasia Battery-Revised WAB-R [33]. As part of the initial assessment, hearing screenings were conducted to ensure participants were able to hear experimental stimuli. The hearing screening consisted of frequency sweeps between 250–8000 Hz and between 30–60 dBs. As this report is part of a larger study, IWA were also administered the SOAP Test of Sentence Comprehension [34] to identify sentence comprehension deficits, which were defined as at- or below-chance performance on comprehension of sentences with non-canonical word order (object-relatives and passives). IWA were included if they met clinical consensus for diagnosis and also demonstrated comprehension deficits. All participants were tested at San Diego State University and were compensated for their time. This study was approved under both the University of California San Diego and San Diego State University’s IRB protocols.

### 2.2. Stimuli

To explore the effect of semantic bias on processing effort, semantic relatedness of the adjective modifying the head (target) noun was manipulated. As shown in Table 2, this manipulation resulted in two conditions: semantically biased adjective (i.e., venomous snake) and unbiased adjective (i.e., voracious snake).

Stimuli were taken from Akhavan et al. [13]. As we were interested in the effects of semantic biasing of an adjective towards a noun, the experimental sentences contained either a biased adjective–noun pair or an unbiased adjective–noun pair. The adjectives were matched for syllable length, but, as determined by pretesting (described in Akhavan et al., 2022 [13]), the unbiased adjectives had low semantic value and the biased adjectives had high semantic value. Sentences were recorded by an adult female Native English speaker, with an average speaking rate of 4.47 syllables per second (see Appendix A for full list of sentence stimuli).

**Figure 1:**
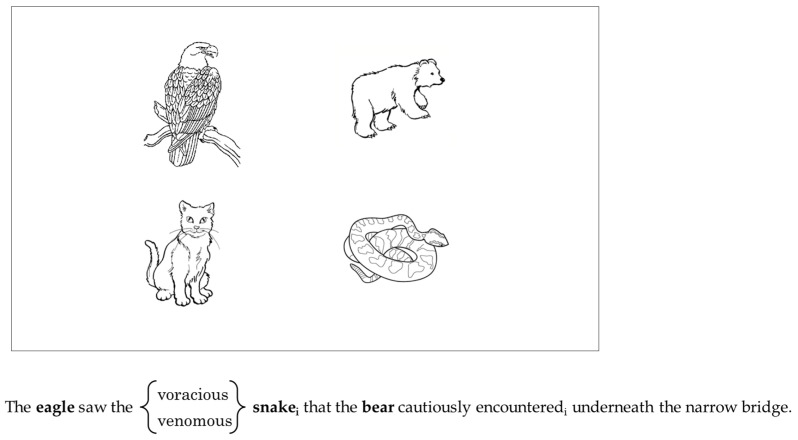
Example of the visual world display and a sample experimental sentence.

The images used in this study were simple black-and-white line drawings of animals (see Figure 1). They were all 450 × 450 pixels and were presented in a 2 × 2 visual display. Three of the images corresponded to a noun in the sentence, while the fourth was a distractor image.

Then, 60 experimental sentences (30 with a semantically biased adjective and 30 with a semantically unbiased adjective) were combined with 60 control sentences, for a total of 120 sentence stimuli. Control sentences were canonical, subject-relative constructions containing multiple nouns (e.g., “A few days ago at the book release party, the author that observed the illustrator quickly escaped after the opening remarks”). Following Akhavan et al. [13], 3 of the 60 experimental sentences were excluded from analysis (2 were removed because the pretest data did not reveal a clear semantic bias and 1 because the unbiased adjective was found to be slightly biased towards another noun in the sentence. Thus, data from 57 of the experimental sentences were moved forward for analysis and will be discussed below.

### 2.3. Procedure

Using an eye-tracking-while-listening with a visual world paradigm, pupil size (current study) and gaze location [13] were measured during real-time auditory sentence processing. Pupil and gaze data were collected using a Tobii X-120 eye-tracker (Tobii, Stockholm, Sweden) with a 60 Hz sampling rate. In this within-subjects design, all sentences were divided into four scripts of 30 items each. Conditions were counterbalanced across scripts such that each sentence only appears once per condition per visit. Data were collected over the course of 4 sessions, with at least one week in-between visits. Visits and conditions were counterbalanced across participants.

**Figure 2:**
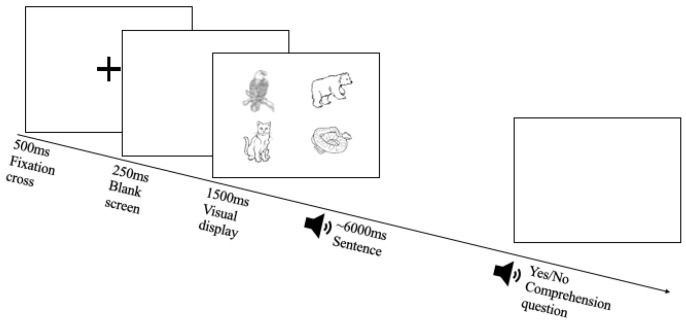
An example of the experimental procedure. Each trial would begin with a fixation cross, followed by a blank screen, and then the 2 × 2 image display. Following the onset of the visual display, sentences were presented over headphones while participant eye gaze and pupillary data were collected. At the end of each trial, participants were asked a comprehension question to ensure they were attending to the auditory information.

Participants listened to sentences over headphones while viewing the four images on the screen. Participants were situated in front of the computer screen with an attached Tobii X-120 eye-tracker with a distance of 60 cm between their eyes and the eye-tracker. The eye-tracker was calibrated at the beginning of each experimental session. Across each trial, change in pupillary diameter was sampled at a rate of 60 Hz, recording about every 17 ms. Stimuli were presented using E-Prime 2.0 software [35]. For every trial, a fixation cross was presented for 500 ms to give the participant time to fixate and adjust, followed by a blank screen for 250 ms. Then, the image display was presented for 1500 ms before the auditory sentence began and remained on screen for 500 ms after the end of the sentence. Presentation of auditory sentences lasted around 6000 ms while visual stimuli remained on the screen. To keep participants on track, comprehension questions were administered where participants were asked a yes–no question after each trial (e.g., “Was the bear under the narrow bridge?”), responding via a button box using their left, non-paretic hand (see Figure 2 for a depiction of the procedure). Each experiment began with 10 practice trials to confirm understanding of the task.

### 2.4. Preprocessing

#### 2.4.1. Data Cleaning

Data were preprocessed using the pupillometry portion of the GazeR package, v0.0.1.2 [26] in R [36]. Since the GazeR package addresses pupil data in a visual world paradigm, it was best suited for this data set. We followed Geller et al.’s [26] recommendations for preprocessing settings. The data processing steps include de-blinking, smoothing and interpolating, baseline correction, artifact rejection, and median absolute deviation, as described below.

Pupillometry (absolute pupil diameter) data were collected using the Tobii X-120 Eye-tracker, which records absolute pupil diameter separately from gaze data. Mean pupil diameter was calculated for the left and right eyes (if data were available for both). If only one eye had data due to recording error in the other eye, that value was used for further analysis (see [37], for a similar procedure). Next, since blinks can distort the pupil size as the eyelids open and close, the blinks automatically recorded by the eye-tracker were coded as “NA” and the blink function was used to extend data around the blink by 100 ms on either side. We applied linear interpolation and then performed a smoothing operation using a 5-point moving average [26]. Next, we followed a baselining procedure to normalize pupil size across trials. This process of baselining has been a recent topic of discussion in the pupillometry literature as there can be differences in results depending on whether the chosen procedure uses linear or non-linear correction [38]. Based on current consensus, we adopted the subtractive baseline correction protocol (corrected pupil size = pupil size − baseline), a linear procedure, which is argued by Mathôt et al. [39] to be more robust and resistant to distortions compared to other corrective protocols.

#### 2.4.2. Data Loss

Based on Winn et al.’s [40] best practice for pupil data cleaning, only participants with less than 20% data loss were included in the analysis. Reasons for data loss include excess blinking, participants looking away, and loss of data due to technical failure. Given these criteria, data from 4 AMC and 2 IWA were removed from further analysis. The remaining 16 participants that were included in the analysis included 7 AMC and 9 IWA. Finally, we created time bins (100 ms; following [26]) across the whole sentence via the downsample function.

#### 2.4.3. Time Windows for Analysis

**Figure 3:**
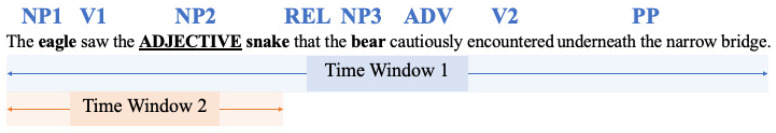
Time windows of interest. Time Window 1 includes the whole sentence. Time Window 2 is from the beginning of the sentence until the offset of NP2.

Two time windows of interest were identified across the whole sentence (Figure 3). The pupil response was time-locked to the specific point in a sentence in which the experimental manipulation was hypothesized to influence processing load. The first window of interest was the whole sentence to capture global effects on pupillary response. To explore effects of experimental condition on initial processing of the target noun (NP2), which appears just after the semantically biased or unbiased noun, the second window of interest began at sentence onset and ended at the offset of the second noun.

#### 2.4.4. Growth Curve Analysis

Growth curve analysis is a method of analyzing change over time through fitting orthogonal polynomial curves over time course data [41,42]. These models allow us to analyze the change in pupil dilation response over time, and, thus, draw conclusions about the change in processing effort over the course of the time window. This is a popular method for analyzing gaze data and has been applied to pupil data previously, i.e., [43]. For Time Window 1, three orthogonal polynomials were applied to capture the complex morphology. For Time Window 2, two orthogonal polynomials were applied (see Table 3 for further explanation of data fit terms).

The mean pupil response was the dependent variable, and the variables of interest were differences between Group and differences between Bias Condition. All analyses were completed using the statistical software R-3.2.1 [36] in R-Studio, with the LmerTest package, v3.1-3 [44]. To capture individual and trial level variability, random effects for subject and trial were added on the intercept and slope into the growth curve models. *p*-values were calculated using Satterthwaite’s approximation for degrees of freedom.

## 3. Results

### 3.1. Comprehension

As previously stated, participants responded to yes/no questions after each trial, to ensure they paid attention to the elements in each sentence. Response data were submitted for analysis using a mixed-effects logistic regression model to explore group and condition differences. As anticipated, the results revealed an effect of the group (AMC and IWA); specifically, the IWA group performed worse than the AMC group (Estimate = −1.12, SE = 0.23, *p* < 0.05). No effect of the condition was found for accuracy within the AMC or IWA group (See Table 4). Given the complexity of the sentences combined with the task, it is unsurprising that participants underperformed. Importantly, however, the participants in both groups made the binary decisions throughout the task. As was the case in the prior work [13], these offline data were not used to inform the online analysis.

### 3.2. Pupil Dilation Response Related to Group Effects—Time Window 1

**Figure 4:**
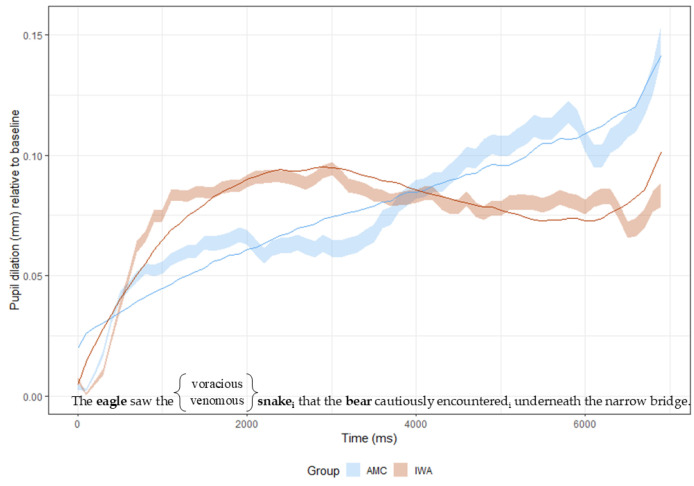
Time Window 1. Pupil responses throughout the whole sentence for AMC (blue) and IWA (red). Data are indicated by the shaded ribbons and growth curve models are indicated by the solid line.

The first set of analyses assessed the processing effort associated with the whole sentence for auditorily presented complex sentences (object-relative constructions) for both groups of participants. Figure 4 shows the time course of changes in pupillary reaction (in millimeters) for both IWA and AMC while listening to the object-relative sentence constructions.

To evaluate the change in pupil size across a whole sentence, third-order (cubic) orthogonal polynomials were applied. Each of the terms represent different aspects of the response—see Table 3 adapted from [43,45].

Analyses of pupil dilation response across the whole sentence, averaged across conditions, revealed a significant main effect of Group (χ^2^ = 11.40, *p* = 0.02), such that IWA and AMC show different patterns throughout the course of the sentence (see Figure 4). This resulted in a significant quadratic term (Estimate = −0.10, SE = 0.04, t = −2.578, *p* = 0.02), which signifies a group difference in the rise and fall of the pupil reaction, indicating that AMC showed a shallower curve compared to IWA. This also resulted in a significant cubic term (Estimate = 0.07, SE = 0.026, t = 2.782, *p* = 0.013), which highlights the difference in timing of the peak pupil response, such that the AMC displayed an earlier inflection point than IWA. Table 5 shows the full results of this analysis.

As depicted in Figure 4, there were clear visual differences in the pupillary response pattern between the AMC and IWA groups at different points in the sentence. The AMC group (blue line in Figure 4) shows an overall increase in pupil diameter, with pockets of pupil size reduction. As the AMC participants begin to build a syntactic structure upon hearing the first NP (the eagle), the pupil response increases and remains somewhat steady until around 3000 ms (about when the third noun, the bear, is heard). At that moment in the sentence (i.e., around 3000–3500 ms), there is an inflection point indicating a shift in the trajectory of the pupillary response (this pattern is captured by the cubic time term). After processing the verb (encountered), the pupillary response appears to dip briefly, and then continues to rise until the end of the sentence.

The pattern described for the AMC group differs from what is seen for IWA (red line, Figure 4). Unlike the AMC group, IWA do not show a continual increase in pupillary response—possibly indicating a plateau in processing effort (see Discussion for more details). While the IWA evince an initial peak in pupillary response upon hearing the first noun (‘the eagle’) similar to the AMC group, this initial increase surpasses that of the AMC group, indicating more processing effort at the beginning of the sentence. As the sentence continues to unfold, unlike the AMC group, the initial increase in pupillary response plateaus. There is no modulation in pupillary response at the relativizer (‘that’) or the main verb (‘encountered’), which are interpreted here as reflecting a “cognitive overload” [46]. This lack of an effect is described in detail in the Discussion section.

### 3.3. Pupil Dilation Response Related to Semantic Biasing—Time Window 2

**Figure 5:**
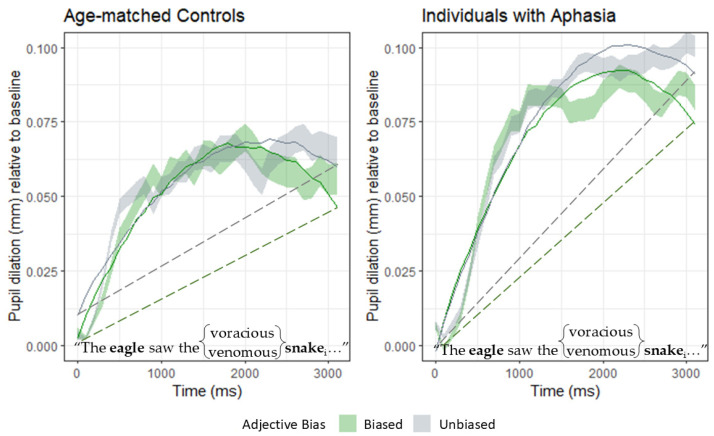
Time Window 2. Pupil responses from the beginning of the sentence to the onset of the second noun. AMC data are graphed on the left, IWA data on the right. The biased adjective condition for each group is shown in green, and the unbiased adjective condition is shown in grey. Data are indicated by the shaded ribbons and growth curve models are indicated by the solid line. Dotted lines were manually inserted to demonstrate visual differences in linear fit.

To examine the direct effect of adjective bias on the processing effort of lexical-level activation, we focused on the time window from the beginning of the sentence up until the offset of the second noun phrase (as shown in Figure 5). As was determined in the overall model for Time Window 1, AMC and IWA have different curve morphologies. Thus, in Time Window 2, we examined each group separately and applied second-order orthogonal polynomials to investigate the rate of change in pupil response.

In the first model, we looked at the condition effect (biased vs. unbiased) for AMC (left panel). As shown by the overlapping green (biased) and gray (unbiased) lines, the AMC begin processing the sentence similarly in both conditions. However, as the adjective is processed, the pupil response curves begin to separate, such that the biased condition results in a continual decrease in pupillary response. The analysis revealed an effect of the condition (χ^2^ = 15.07, *p* = 0.002), which resulted in significant linear (Estimate = 0.01, SE = 0.005, t = 2.071, *p* = 0.038) and quadratic (Estimate = 0.016, SE = 0.005, t = 3.049, *p* = 0.002) terms. When looking from the beginning of the sentence to the end of the target noun, the quadratic effect reveals less of an incline (shallower slope) in the biased condition compared to the unbiased condition. These findings indicate that the semantically biased adjective reduced the processing effort compared to the unbiased adjective.

For the IWA (right panel), they showed a similar pupil response at the beginning of the sentence for both conditions, but the conditions separate during the processing of the adjectives. Across Time Window 2, a condition effect (χ^2^ = 57.88, *p* < 0.001) is captured by a significant linear term (Estimate = 0.03, SE = 0.004, t = 7.229, *p* < 0.001), indicating an overall shallower slope (and, therefore, reduced processing effort) in the biased condition as compared to the unbiased condition.

Unlike the AMC group, IWA only showed a trend towards a difference in the shape of the curve (quadratic term) in the biased condition. The analysis revealed that the quadratic term for the IWA approached, but did not reach, significance (Estimate = 0.008, SE = 0.004, t = 1.895, *p* = 0.058). See Table 6 for the full results of this analysis.

## 4. Discussion

This study explored how age-matched controls and individuals with aphasia expend processing effort at the lexical level during complex sentence processing through the pupillary response. Specifically, we tested whether semantic cueing, through adjective biasing, reduces the processing effort during lexical activation and whether a reduced processing effort improves sentence processing overall. What emerged, and is not surprising, is that the two groups utilized the semantic cues differently throughout and within the sentence.

Age-matched controls demonstrated an upward trend of processing effort across the sentence. These findings support other pupillometry studies [43] that demonstrate pupil size generally increases throughout the task as greater cognitive engagement is required. This suggests that the increase in pupil response in AMC stems from the effort involved in incrementally parsing and building the structure over time. When examining the effect of semantic biasing on noun processing, AMC began to show a decrease in processing effort in the semantically biased condition during the adjective.

In contrast to the AMC group, IWA showed marked differences in processing effort across the sentence. The pupillary response plateaus just after the target noun, suggesting that the IWA failed to build the sentence structure. These results are in line with research that suggests that IWA have difficulty processing object-relative sentences, i.e., [4]. Interestingly, IWA did demonstrate semantic sensitivity to the biased adjective. Data revealed reduced processing effort in the biased condition compared to the unbiased condition. What follows is a discussion on the observed pupillary patterns in both groups and how those patterns correspond to their gaze patterns in Akhavan et al. [13].

### 4.1. Processing Effort During Complex Non-Canonical Sentence Processing

Recall that we found significant group differences in the pupil response between AMC and IWA at Time Window 1. Similar to prior reports, we found differences between AMC and IWA in processing at the sentence level [18], but not at the target noun [28,29].

Interestingly, similar to the AMC group, IWA did demonstrate incremental increases in processing effort at the beginning of the sentence. Unlike AMC, however, this increase in cognitive effort was not sustained throughout the sentence. This is intriguing because these complex object-relative sentence constructions require a significant amount of structure building. Instead, the IWA show a plateau in pupillary response around the point when the syntactically driven reactivation of the direct object (verb offset) is required. This plateau in pupil response is characteristic of a “cognitive overload” [46]. Cognitive overload is a phenomenon observed in the pupillometry literature, e.g., [47,48], in which the amount of effort required in a task exceeds the current capacity of the participant’s cognitive resources [49]. For example, in a digit span task, this can result in the pupillary response reaching a maximum dilation and then constricting once the working memory capacity has been reached—what van der Wel and van Steenbergen [49] refer to as reaching an asymptote, and what we have described in this study as a plateau in pupillary response. As IWA typically have impairments with processing syntactic dependencies in object-relative sentences, it is likely that the observed plateau in pupil response reflects a disengagement from the task and offline comprehension failure. As suggested in Chapman and Hallowell [18], it is possible that IWA have difficulty adequately allocating cognitive resources during language processing, and thus reach a capacity limitation when attempting to process object-relative sentences. While we believe it is important to acknowledge such a striking pattern, we have limited this discussion about cognitive overload as it is beyond the scope of this study, which focuses on initial lexical access.

### 4.2. Effect of Semantic Biasing on Noun-Processing Effort

When looking closely at the second time window to investigate the immediate effect of a semantically biased adjective on the subsequent noun, we found that, for both AMC and IWA, the effect of the adjective biasing was present in both groups such that the biased condition resulted in a smaller pupillary response. Our findings support the notion that semantic biasing may reduce the processing effort involved in lexical–semantic retrieval for both groups at the initial processing of the target noun. Here, we found that, like other methodological manipulations (word frequency [50,51], neighborhood density [52], and cognate status [53]), the pupillary response can detect the sensitivity to lexical–semantic manipulations while processing sentences in real time.

Recall that we proposed three possible theoretically motivated outcomes for IWA pupillary responses: (1) no pupillary response to semantic biasing; (2) a timely pupillary response to semantic biasing; and (3) a delayed pupillary response to semantic biasing. The current pupil data support previous findings that IWA have a sensitivity to semantic information in the lexical system [28], despite demonstrating deficits in the time course of lexical activation and/or integration as observed through alternative methods [3,4,13,14,15]. Thus, while these data cannot lend support to the lexical integration deficit hypothesis, they do provide evidence for the real-time sensitivity to the semantic network. As a reminder, the data presented here were collected simultaneously with the gaze data reported in Akhavan et al. [13]. In the Akhavan et al. [13] report, no effect of bias was found on the gaze patterns for the IWA group at the initial target noun. Given the current findings from this paper, it is clear that the gaze data did not capture the full story. It may be the case that pupillometric responses are better able to indicate the intactness of a semantic network when compared to other real-time measures, such as gaze data from eye-tracking methods. Our pupil data add to this story by assessing lexical–semantic activation through the lens of cognitive processing effort. The current data reveal that IWA are sensitive to semantic cues, but, taken together with the gaze data from Akhavan et al. [13], IWA are not able to integrate that information in real time to facilitate access of the target noun. This begs the question—how can IWA be ‘sensitive’ to lexical–semantic cues enough to show a biasing effect, yet are unable to integrate the actual forms into their parsing system?

We argue that the different measures of eye-tracking while listening (gaze and pupillary response) reflect different levels of processing. Eye gaze responses, as collected in Akhavan et al. [13], are indicative of lexical activation and sentential integration. We propose that the pupil response measures a form of lexical–semantic processing that is underlying the surface representations—that is, pupil responses are indexing the implicit cognitive–linguistic processing associated with lexical activation. From the pupillometry literature in cognitive neuroscience, the pupil response is tied to the noradrenergic system, which is modulated by the locus coeruleus [16]. This is widely thought of as a domain-general arousal mechanism, which feeds multiple cognitive functions such as memory and attention. There is evidence in the literature that implicates the system of the unconscious processing of information [37]. As further discussed in Rojas et al. [54], the pupillary response may capture cognitive processes that are partially activated but are not at the level of processing needed to direct eye gaze during sentence processing or for overt behaviors associated with final comprehension, though more standardized pupillometric studies should be conducted [54]. Further research should seek to disentangle the levels of the pupillary and gaze response to understand the role of the processing effort during lexical activation, and, specific to this study, should seek to increase the sample size to allow for individual-level analyses.

## 5. Conclusions

From this exploratory study on the pupillary response to lexical–sematic cues during sentence processing in aphasia, we have found evidence to suggest that individuals with aphasia are responsive to lexical–semantic cues despite impaired real-time lexical access. We have also demonstrated that individuals with aphasia show impaired effort in structure building while processing complex sentences. These findings highlight the importance of considering lexical–semantic cue responsiveness and processing effort dynamics on lexical-level activation during sentence processing in aphasia.

## Figures and Tables

**Table 1:** Individuals with aphasia demographics.

IWA	Sex	Years Post-Stroke	Age at Testing	Years of Education	Lesion Location	BDAE-3 Severity Level	WAB-AQ	SOAP-SR (%)	SOAP-OR (%)
009	M	15	55	17	L IFG (BA44 and BA45) and PFG w/subcortical extension; w/sparing of the occipital lobe	4	67.7	60	40
017	M	18	66	15	L ACA and MCA infarct	4	95.4	100	90
101	M	9	67	20	L IFG (BA44) and PFG w/subcortical extension; w/sparing of the occipital lobe	2	82.6	100	30
130	M	8	63	16	L IPL w/posterior extension sparing STG	4	90.5	75	55
140	F	16	42	16	L MCA infarct	2	75.7	80	30
151	F	7	65	16	L MCA infarct w/subcortical extension	4	95.8	100	100
159	F	6	64	16	L MCA infarct	3	92.4	100	70
165	F	4	64	12	L MCA infarct	3	ND	80	60
169	M	4	59	12	L MCA infarct	2	28.2	80	40
190	F	6	76	12	L STG	3	88.2	90	40
191	M	1	57	16	L MCA infarct	4.5	98.4	100	60

M = male, F = female; L = left; BA = Brodmann area; IFG = inferior frontal gyrus; PFG = posterior frontal gyrus; IPL = inferior parietal lobule; STG = superior temporal gyrus; ACA = anterior cerebral artery; MCA = middle cerebral artery; BDAE = Boston Diagnostic Aphasia Examination (0 = no usable speech or auditory comprehension, 5 = minimal discernable speech handicap); WAB-AQ = Western Aphasia Battery Aphasia Quotient (0 = very severe, 76+ = mild); SOAP SR = average percent correct on subject-relative items from the SOAP Test of Auditory Sentence Comprehension; SOAP OR = average percent correct of object-relative items from the SOAP Test of Auditory Sentence Comprehension; ND = No data available.

**Table 2:** Example of experimental sentence constructions.

Semantic Condition	Example Sentence
Biased adjective	The eagle saw the **venomous snake_i_** that the bear cautiously encountered_i_ underneath the narrow bridge.
Unbiased adjective	The eagle saw the **voracious snake_i_** that the bear cautiously encountered_i_ underneath the narrow bridge.

**Table 3:** Description of time terms from growth curve analysis.

Data Fit Term	What the Term Represents
Intercept	Average pupil size across the time-window
Linear	Constant rate of change over time (i.e., slope)
Quadratic	Second-order orthogonal polynomial. The rate of increase (rise) or decrease (fall) around the primary curve inflection point
Cubic	Third-order orthogonal polynomial. The extent to which there is a secondary inflection point in the response (positive values indicate that the pupil response had an earlier rise and fall, whereas a negative value indicates a later peak)

**Table 4:** Offline comprehension results in terms of percent correct.

	Control	IWA
Biased	77.8%	60.6%
Unbiased	79.3%	61.4%

**Table 5:** Time Window 1 model results. Significant values are bolded.

	**Model fit**		
	χ^2^	*p*	
Base			
Group	11.40	**0.02**	
Group × Condition	4931.92	**<0.001**	
	**Group effect**		
	β	t	*p*
Intercept	0.003	0.119	0.907
Linear	−0.127	−1.199	0.248
Quadratic	−0.104	−2.578	**0.020**
Cubic	0.07	2.782	**0.013**
	**Group × Condition effect**		
	β	t	*p*
Intercept	0.009	0.589	0.564
Linear	0.099	2.362	**0.031**
Quadratic	0.018	0.337	0.741
Cubic	−0.005	−0.118	0.907

**Table 6:** Time Window 2 model results. Significant values are bolded.

	**Model fit**					
	**AMC**			**IWA**		
	χ^2^	*p*		χ^2^	*p*	
Base						
Condition	15.07	**0.002**		57.882	**<0.001**	
	**Condition effect**					
	**AMC**			**IWA**		
	β	t	*p*	β	t	*p*
Intercept	0.009	0.794	0.431	0.004	0.600	0.551
Linear	0.011	2.071	**0.038**	0.031	7.229	**<0.001**
Quadratic	0.016	3.049	**0.002**	0.008	1.895	0.058

## Data Availability

The data used to support the findings of this publication will be made available by the authors upon request, due to privacy reasons.

## Appendix A

**Table A1:** The experimental sentence stimuli used in this study, with the unbiased condition listed in the left column and the biased condition listed in the right column. Adjectives are in bolded font. These sentence stimuli were developed and used in Akhavan et al. [13]. The experimental sentence stimuli used in this study, with the unbiased condition listed in the left column and the biased condition listed in the right column. Adjectives are in bolded font. These sentence stimuli were developed and used in Akhavan et al. [13].

Unbiased Adjective	Biased Adjective
The duck followed the **perfect** kitten that the cow deliberately nudged across the grassy meadow.	The duck followed the **playful** kitten that the cow deliberately nudged across the grassy meadow.
The veterinarian greeted the **popular** king that the criminal mistakenly expected at the stunningly lavish gala.	The veterinarian greeted the **powerful** king that the criminal mistakenly expected at the stunningly lavish gala.
The scorpion annoyed the **anxious** bull that the bee constantly pestered in the abandoned railroad yard.	The scorpion annoyed the **angry** bull that the bee constantly pestered in the abandoned railroad yard.
The crocodile spied the **weird** owl that the chameleon momentarily faced in the exotic animal show.	The crocodile spied the **wise** owl that the chameleon momentarily faced during the exotic animal show.
The crab helped the **coy** puppy that the rabbit relentlessly teased before playful tussle.	The crab helped the **cute** puppy that the rabbit relentlessly teased before the playful tussle.
The lawyer visited the **forgetful** gymnast that the butler allegedly helped with the illegal cover-up.	The lawyer visited the **flexible** gymnast that the butler allegedly helped with the illegal cover-up.
The magician passed the **redheaded** nun that the mailman compassionately soothed after the traumatic event.	The magician passed the **religious** nun that the mailman compassionately soothed after the traumatic event.
The ladybug observed the **smelly** bat that the opossum deliberately avoided near the historic monument.	The ladybug observed the **scary** bat that the opossum deliberately avoided near the historic monument.
The astronaut approached the **sad** jockey that the salesman incorrectly judged throughout the dinner party.	The astronaut approached the **short** jockey that the salesman incorrectly judged throughout the dinner party.
The otter spotted the **shiny** octopus that seagull unsurprisingly smelled after the hot and sunny day.	The otter spotted the **slimy** octopus that seagull unsurprisingly smelled after the hot and sunny day.
The deer noticed the **male** gorilla that the hummingbird thoroughly amused with the acrobatic display.	The deer noticed the **mean** gorilla that the hummingbird thoroughly amused with the acrobatic display.
The ostrich recognized the **delightful** toucan that the baboon hesitantly touched during the bizarre encounter.	The ostrich recognized the **colorful** toucan that the baboon hesitantly touched during the bizarre encounter.
The spider scared the **live** rooster that the porcupine accidentally bumped on the side of the country road.	The spider scared the **loud** rooster that the porcupine accidentally bumped on the side of the country road.
The dentist helped the **tired** maid that the plumber heartlessly cheated in spite of the cautious investment.	The dentist helped the **tidy** maid that the plumber heartlessly cheated in spite of the cautious investment.
The orangutan examined the **defenseless** cockroach that the parrot quickly located near the bottom of the staircase.	The orangutan examined the **disgusting** cockroach that the parrot quickly located near the bottom of the staircase.
The parrot examined the **pretty** dove that the orangutan casually shoved off of the exam table.	The parrot examined the **peaceful** dove that the orangutan casually shoved off of the exam table.
The salesman approached the **slim** wrestler that the astronaut unconditionally loved in spite of the tabloid gossip.	The salesman approached the **strong** wrestler that the astronaut unconditionally loved in spite of the tabloid gossip.
The baboon recognized the **shy** tortoise that the ostrich understandably scared with the unexpected noise.	The baboon recognized the **slow** tortoise that the ostrich understandably scared with the unexpected noise.
The chameleon spied the **plump** flamingo that the crocodile instinctively grabbed during the calculated ambush.	The chameleon spied the **pink** flamingo that the crocodile instinctively grabbed during the calculated ambush.
The bee annoyed the **miniature** unicorn that the woodpecker distinctively heard at the end of the fairy tale.	The bee annoyed the **magical** unicorn that the woodpecker distinctively heard at the end of the fairy tale.
The porcupine scared the **puny** skunk that the spider unexpectedly bit under the deck in the back yard	The porcupine scared the **putrid** skunk that the spider unexpectedly bit under the deck in the back yard.
The mailman visited the **humorous** queen that the magician easily confused in the middle of the fundraiser.	The mailman visited the **homecoming** queen that the magician easily confused in the middle of the fundraiser.
The opossum observed the **grimy** swan that the ladybug lazily circled in the pond at the city park.	The opossum observed the **graceful** swan that the ladybug lazily circled in the pond at the city park.
The rabbit helped the **sleepy** rat that the crab repeatedly pinched during the minor scuffle.	The rabbit helped the **sneaky** rat that the crab repeatedly pinched during the minor scuffle.
The butler visited the **happy** prince that the lawyer properly instructed in the art of negotiation.	The butler visited the **handsome** prince that the lawyer properly instructed in the art of negotiation.
The criminal greeted the **gabby** mechanic that the veterinarian angrily informed of the fabricated charges.	The criminal greeted the **greasy** mechanic that the veterinarian angrily informed of the fabricated charges.
The plumber helped the **easygoing** cheerleader that the dentist recently met before the city council meeting.	The plumber helped the **energetic** cheerleader that the dentist recently met before the city council meeting.
The hummingbird noticed the **fake** cheetah that the deer cautiously examined next to the perfectly clear lake.	The hummingbird noticed the **fast** cheetah that the deer cautiously examined next to the perfectly clear lake.
The seagull spotted the **incredible** dolphin that the otter eventually found in amongst the dense kelp forest.	The seagull spotted the **intelligent** dolphin that the otter eventually found in amongst the dense kelp forest.
The cow followed the **dizzy** pig that the duck confidently led along the winding country road.	The cow followed the **dirty** pig that the duck confidently led along the winding country road.
